# Visual Functions Affecting Vision-Related Quality of Life Following Intravitreal Ranibizumab Therapy for Central Retinal Vein Occlusion

**DOI:** 10.3390/jcm11144139

**Published:** 2022-07-16

**Authors:** Tomoya Murakami, Fumiki Okamoto, Yoshimi Sugiura, Shohei Morikawa, Yoshifumi Okamoto, Takahiro Hiraoka, Tetsuro Oshika

**Affiliations:** 1 Department of Ophthalmology, Faculty of Medicine, University of Tsukuba, 1-1-1 Tennoudai, Tsukuba 305-8575, Ibaraki, Japan; fumiki-o@md.tsukuba.ac.jp (F.O.); yoshimis@md.tsukuba.ac.jp (Y.S.); s0711715@yahoo.co.jp (S.M.); y-okamoto@md.tsukuba.ac.jp (Y.O.); thiraoka@md.tsukuba.ac.jp (T.H.); oshika@eye.ac (T.O.); 2 Mito Kyodo General Hospital, 3-2-7 Miyamachi, Mito 310-0015, Ibaraki, Japan

**Keywords:** vision-related quality of life, cystoid macular edema, central retinal vein occlusion, intravitreal injection, ranibizumab, best-corrected visual acuity

## Abstract

Visual functions that affect vision-related quality of life (VR-QoL) before and after intravitreal injection of ranibizumab in patients with cystoid macular edema secondary to central retinal vein occlusion (CRVO-CME) are poorly understood. This multicenter, open-label, single-arm prospective study included 23 treatment-naïve patients with CRVO-CME. The best-corrected visual acuity (BCVA), letter contrast sensitivity (LCS), severity of metamorphopsia (M-CHARTS), amount of aniseikonia (New Aniseikonia Test), and stereopsis (Titmus Stereo Test and TNO stereotest) were examined every month from before treatment to 12 months after treatment. For VR-QoL assessment, the 25-item National Eye Institute Visual Function Questionnaire (VFQ-25) was provided to the patients before treatment and at 3, 6, and 12 months after treatment. Stepwise multiple regression analysis revealed that the BCVA of the fellow eye was related to the VFQ-25 composite score before treatment, and that the BCVA of the fellow eye and TNO values were related to the VFQ-25 composite score 12 months after treatment. Changes in LCS were significantly correlated with changes in the VFQ-25 composite score. In patients with CRVO-CME, visual acuity of the fellow eye had the strongest impact on VR-QoL. The contrast sensitivity of the affected eye and stereopsis were also associated with VR-QoL.

## 1. Introduction

Cystoid macular edema secondary to central retinal vein occlusion (CRVO-CME) is a common vision-threatening complication in patients with central retinal vein occlusion (CRVO) [1]. The advent of anti-vascular endothelial growth factor (VEGF) therapy has improved the visual prognosis of patients with CRVO-CME [2,3]. However, even if CME resolves after treatment, some patients may not have satisfactory visual function due to metamorphopsia [4], decreased contrast sensitivity (CS) [5], and decreased stereopsis [6].

Visual functions are assessed using visual acuity (VA) tests, and VA is considered an important determinant of vision-related quality of life (VR-QoL). However, visual functions other than VA are also related to VR-QoL in some vitreoretinal diseases. For example: the severity of metamorphopsia and aniseikonia, not VA, correlates with VR-QoL in patients with epiretinal membrane (ERM) [7,8]; the severity of metamorphopsia, not VA, correlates with VR-QoL in patients with macular hole (MH) [9]; and CS and stereopsis, not VA, correlate with VR-QoL in patients after rhegmatogenous retinal detachment (RRD) surgery [10].

Several researchers have investigated visual functions other than VA in patients with CRVO-CME. Manabe et al. investigated the incidence of metamorphopsia in patients with CRVO-CME after anti-VEGF therapy and reported that the percentage of patients with metamorphopsia did not decrease with treatment (50% at baseline and 57% 6 months after treatment) [4]. Murakami et al. investigated CS in patients with CRVO-CME after anti-VEGF therapy and reported that the CS of the affected eye improved after treatment, although it remained worse than that of the fellow eye. The authors further found that CS was associated with VR-QoL after treatment [5]. Okamoto et al. investigated stereopsis in patients with CRVO-CME after anti-VEGF therapy and reported that stereopsis improved after treatment. However, stereopsis remained worse than that in individuals without ocular disease and was associated with VR-QoL at baseline and after treatment [6]. Deramo et al. reported that the best-corrected VA (BCVA) of the fellow eye was more related to VR-QOL than the VA of the affected eye in patients with CRVO-CME [11]. To date, however, it is unclear which visual function is most important for VR-QoL in patients with CRVO-CME.

Therefore, in this study, we aimed to determine which of the visual functions—VA, CS, metamorphopsia, aniseikonia, and stereopsis—primarily affect VR-QoL in patients with CRVO-CME following anti-VEGF therapy.

## 2. Materials and Methods

This multicenter, open-label, single-arm prospective study was approved by the Institutional Review Board of Tsukuba University Hospital and Mito Kyodo General Hospital and was conducted in accordance with the tenets of the Declaration of Helsinki. Informed consent was obtained from all subjects involved in the study.

This study included patients who were diagnosed with treatment-naïve CRVO-CME at Tsukuba University Hospital or Mito Kyodo General Hospital between May 2016 and December 2018. Patients with ophthalmic disorders other than mild refractive errors and cataracts were excluded. Additionally, patients with severe diabetes, hypertension, cerebrovascular disease, or ischemic heart disease were excluded.

### 2.1. Assessments

Every month from baseline to 12 months after treatment, the best-corrected VA (BCVA), letter CS (LCS), severity of metamorphopsia, amount of aniseikonia, stereopsis, and retinal microstructure were examined. BCVA and LCS in the fellow eye were examined at baseline. The BCVA was measured using the Landolt chart and was converted to logarithm of the minimum angle of resolution (logMAR) values for calculation.

### 2.2. Contrast Sensitivity

Letter optotypes in the CSV-1000LV chart (Vector Vision, Houston, TX, USA) were used to assess CS. The optotypes are of the same size and have a low spatial frequency (2.4 cyc/deg). The chart comprises 24 letters. There are eight contrast levels (standard, 35.5%, 17.8%, 8.9%, 6.3%, 4.5%, 2.2%, and 1.1%). Each contrast level has three letters. The patient is given a score ranging from 0 to 24, depending on the number of letters he or she can correctly read. LCS was measured monocularly from a distance of 2.5 m with appropriate correction.

### 2.3. Metamorphopsia

M-CHARTS (Inami, Tokyo, Japan) was used to quantify the severity of metamorphopsia. M-CHARTS comprises 1 straight line and 19 dotted lines with dot intervals ranging from 0.2° to 2.0° of the visual angle. The patients viewed M-CHARTS from a distance of 30 cm with appropriate correction. When the straight line is substituted with a dotted line and the dot interval is changed from fine to coarse, the line distortion decreases with the enhanced dot interval until the line appears straight. First, we showed vertical straight lines (0°) to the patient. The severity of metamorphopsia was zero if the patient recognized a straight line as straight. If the patient recognized a straight line as irregular or curved, we proceeded to the next page of M-CHARTS, where the dot intervals of the dotted lines changed from fine to coarse, one after the other. When the patient recognized a dotted line as being straight, the visual angle separating the dots was determined to represent the severity of metamorphopsia for vertical lines. The M-CHARTS was then rotated by 90°, and the test was repeated with horizontal lines. We calculated the average severity of metamorphopsia from the vertical and horizontal severity of metamorphopsia. The examinations were repeated three times, and the mean values were used for data analyses.

### 2.4. Aniseikonia

The New Aniseikonia Test (NAT; Handaya, Tokyo, Japan) was used to evaluate the amount of aniseikonia. Plates with matched pairs of semicircles are required for the NAT (one red and one green). Semicircles of different sizes (diameters vary in 1% of the steps) are arranged in pairs along a series. The plates are viewed from a distance of 66 cm with appropriate correction. Patients wear red/green spectacles and view the plates such that, for each pair of semicircles, one semicircle is seen by the right eye and the other by the left eye. The patient identifies the pair in which both semicircles appear to be of the same size. The percentage of aniseikonia is represented by the size difference between the semicircles in each pair. The degree of aniseikonia in the horizontal and vertical meridians was quantified. For the analysis, we calculated the average amount of aniseikonia from the vertical and horizontal amounts of aniseikonia. The examinations were repeated three times, and the mean values were used for data analyses. A mean aniseikonia of +2% or more was defined as macropsia, whereas a mean aniseikonia of 2% or less was defined as micropsia [12].

### 2.5. Stereopsis

Retinal damage in eyes with CRVO is not limited to the foveal area. Rather, the damage involves a wide area. Therefore, we performed two stereoscopic tests with different index sizes. The Titmus Stereo Test (TST; Handaya, Tokyo, Japan) and TNO stereotest (Handaya) were performed under appropriate spectacle corrections at a standard viewing distance of 40 cm to evaluate stereopsis. The response was verified to ensure that the patient did not use monocular clues during the TST. The results for the TST and TNO were expressed as “seconds of arc” and converted to logarithmic values for statistical analyses.

### 2.6. Vision-Related Quality of Life

To assess VR-QoL, the 25-item National Eye Institute Visual Function Questionnaire (VFQ-25) was provided to all patients before treatment and at 3, 6, and 12 months after treatment. The VFQ-25 consists of questions about general health status and 11 vision-related parameters, including general VA, eye pain, near activity, far activity, social functioning, mental health, role difficulty, dependence, driving, color vision, and peripheral vision. Each parameter is graded on a scale of 0–100. The VFQ-25 composite score is calculated as the unweighted average response to all items, omitting questions on general health [13].

### 2.7. Spectral-Domain Optical Coherence Tomography

We assessed the retinal microstructure using spectral-domain optical coherence tomography (SD-OCT; Cirrus high-definition OCT; Carl Zeiss AG, Jena, Germany). Five-line raster cross-scans were performed using Cirrus analysis software version 3.0. Scans with signal strengths greater than 6/10 were deemed appropriate. We manually measured the central foveal thickness (CFT) using the Cirrus analysis software.

### 2.8. Treatment

Patients were treated using three monthly intravitreal injections of ranibizumab (IVR; 0.5 mg. Lucentis; Novartis Pharma K.K., Tokyo, Japan). After three monthly injections, patients received additional IVR if any of the following retreatment criteria were satisfied: (1) CFT of over 300 μm; (2) new or persistent cystoid retinal changes; (3) subretinal detachment; and (4) subretinal hemorrhage.

### 2.9. Statistical Analyses

We used Wilcoxon’s signed-rank test to assess changes in visual functions (BCVA, LCS, severity of metamorphopsia, amount of aniseikonia, and TST and TNO values), the VFQ-25 composite score, and CFT. Spearman’s rank correlation coefficient was used to assess the relationship between various visual functions and VR-QoL. Multivariate analysis with stepwise regression was used to determine the visual functional parameters that were significantly relevant to the VFQ-25 composite score. All analyses were performed using SPSS version 27 (IBM Corp., Armonk, NY, USA). A *p*-value of <0.05 was considered significant.

## 3. Results

### 3.1. Participants

We included 23 eyes of 23 patients with CRVO-CME in the study. Table 1 lists baseline clinical characteristics. The mean age was 72.2 ± 11.1 years. Of the 23 patients, 13 were men and 10 were women. The mean number of IVR injections was 5.7 ± 2.0. No significant complications, such as endophthalmitis, retinal detachment, or cataracts, were observed. No patients underwent cataract surgery during the study period.

### 3.2. Changes in Visual Functions

Table 2 shows the visual functions, VFQ-25 composite score, and CFT of the patients at baseline and 3, 6, and 12 months after treatment. BCVA, LCS, TST value, TNO values, VFQ-25 composite score, and CFT significantly improved from baseline to 3, 6, and 12 months after treatment, whereas the severity of metamorphopsia and the amount of aniseikonia did not change significantly.

Table 3 shows the results of simple regression analysis and stepwise multiple regression analyses for the correlation between the VFQ-25 composite score and visual functions at baseline. Simple regression analysis revealed that, at baseline, the BCVA of the fellow eye and TST values were significantly correlated with the VFQ-25 composite score. No other visual function was found to be correlated. The results of stepwise multiple regression analyses revealed that the VFQ-25 composite score was significantly associated with the BCVA of the fellow eye.

Table 4 shows the results of simple regression analysis and stepwise multiple regression analyses between the VFQ-25 composite score and visual functions 12 months after treatment. Simple regression analysis revealed that 12 months after treatment, the BCVA and LCS values of the affected and fellow eyes and TNO values were significantly correlated with the VFQ-25 composite score. No other visual function was found to be correlated with the VFQ-25 composite score. The results of stepwise multiple regression analyses revealed that the VFQ-25 composite score was significantly associated with the BCVA of the fellow eye and the TNO value.

Table 5 shows the results of simple regression analysis between changes in the VFQ-25 composite score and visual function before and 12 months after treatment. Changes in the VFQ-25 composite score were significantly associated with changes in LCS values but not with changes in other visual functions.

#### Representative Cases

**Figure 1:**
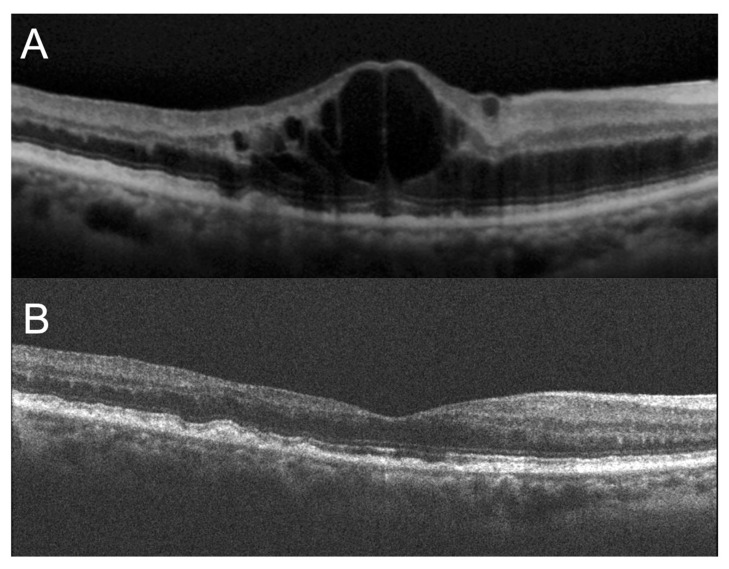
Optical coherence tomography images of an 84-year-old woman with cystoid macular edema secondary to central retinal vein occlusion (CRVO-CME). (**A**) At baseline, CRVO-CME was observed. The logarithm of the minimum angle of resolution (logMAR) best-corrected visual acuity (BCVA) of the affected eye and the 25-item National Eye Institute Visual Function Questionnaire (VFQ-25) composite score were 0.52 and 94.4, respectively. The BCVA of the fellow eye was good (−0.1). (**B**) Twelve months after treatment, macular edema was resolved. The BCVA of the affected eye improved to 0.22. The VFQ-25 composite score was still high (93.8).

The first case is an 84-year-old woman with CRVO-CME (Figure 1). At baseline, the BCVA of the affected eye was poor (0.52); however, the BCVA of the affected eye was good (−0.1), and the VFQ-25 composite score was high (94.4).

**Figure 2:**
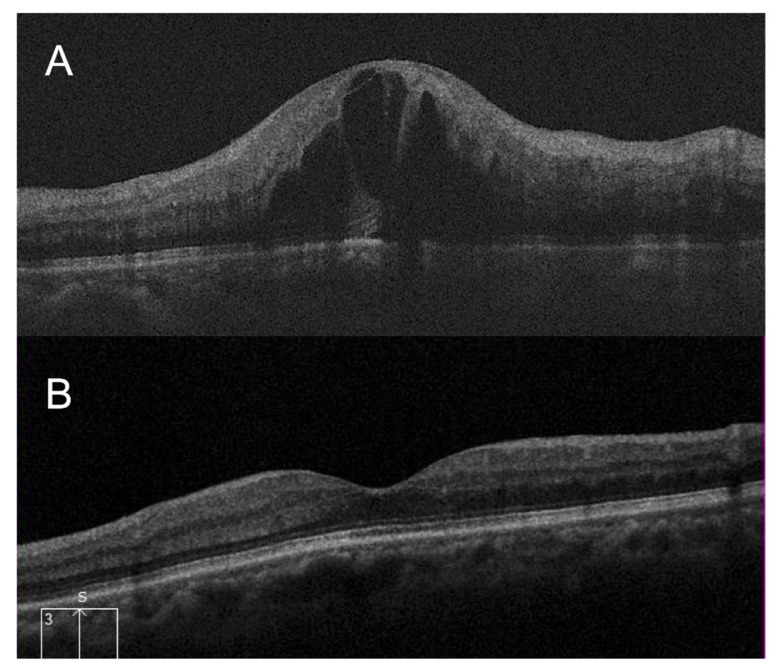
Optical coherence tomography images of a 64-year-old woman with cystoid macular edema secondary to central retinal vein occlusion (CRVO-CME). (**A**) At baseline, CRVO-CME was observed. The logarithm of the minimum angle of resolution (logMAR) best-corrected visual acuity (BCVA), letter contrast sensitivity (LCS) of the affected eye, and the 25-item National Eye Institute Visual Function Questionnaire (VFQ-25) composite score were 0.30, 8 letters, and 52.1, respectively. (**B**) Twelve months after treatment, macular edema was resolved. The BCVA and LCS of the affected eye and the VFQ-25 composite score improved to 0.05, 22 letters, and 88.7, respectively. The improvement in logMAR BCVA was not significant (0.25), but contrast sensitivity and the VFQ-25 composite score significantly improved (14 letters and 36.6, respectively).

The second case is a 64-year-old woman with CRVO-CME (Figure 2). The improvement in logMAR BCVA was not significant (0.25), but contrast sensitivity and the VFQ-25 composite score significantly improved after 12 months of treatment (14 letters and 36.6, respectively).

## 4. Discussion

This study investigated which of the various visual functions is most relevant to VR-QoL in patients with CRVO-CME following anti-VEGF therapy. Stepwise multi-regression analysis revealed that the BCVA of the fellow eye was significantly correlated with the VFQ-25 composite score at baseline and 12 months after treatment. This correlation was stronger than that with any other visual function. Deramo et al. reported that the VFQ-25 scores were associated with BCVA in the better-seeing eye in patients with CRVO [11], which is consistent with our results. Based on the results of the Deramo et al. study and our study, the BCVA of the fellow eye may be the most important visual functional parameter in patients with CRVO. Simple regression analysis revealed that the BCVA of the affected eye was significantly correlated with the VFQ-25 composite score 12 months after treatment, but it was not significantly correlated with the VFQ-25 composite score before treatment. The cause of this discrepancy is unclear. However, we hypothesized that the improvement of BCVA from 0.79 ± 0.56 at baseline to 0.59 ± 0.61 at 12 months after treatment may have contributed to this discrepancy. Okamoto et al. investigated the relationship between visual functions and VR-QoL before and after surgery in patients with proliferative diabetic retinopathy (PDR) [14]. They found that before surgery, the BCVA of the worse-seeing eye was 1.37, and the BCVA of the better-seeing eye was associated with the VFQ-25 composite score. In contrast, the BCVA of the worse-seeing eye was not associated with the VFQ-25 composite score. After surgery, the BCVA of the worse-seeing eye improved to 0.53, and the BCVAs of both eyes were related to the VFQ-25 composite score. Therefore, we considered that in patients with severe retinal diseases such as PDR and CRVO-CME, the BCVA of the fellow eye, not that of the affected eye, is associated with VR-QoL before treatment, and that after treatment, the BCVA of the affected eye improves and is also related to the VR-QoL. Despite the improvement seen in our study, the BCVA of the affected eye remained worse than that of the fellow eye; thus, stepwise multiple regression analysis showed that the BCVA of the fellow eye had a stronger impact on VR-QoL.

In addition, stepwise multi-regression analysis revealed that the TNO value was significantly associated with the VFQ-25 composite score after treatment. Minimal information is available regarding the relationship between stereopsis and VR-QoL in patients with retinal disorders. Ng et al. investigated the relationship between several visual functional parameters and VR-QoL in patients after RRD surgery and reported that stereopsis, not VA, was correlated with VR-QoL [10]. Several researchers have studied the relationship between stereopsis and VR-QoL in patients with cataracts. Fraser et al. [15] and Datta et al. [16] studied the relationship between visual functions and QoL after first-eye cataract surgery and reported that improvement in stereopsis, not VA, was significantly correlated with improvement in VR-QoL. Acosta-Rojas et al. investigated the relationship between visual functions and VR-QoL in patients undergoing bilateral cataract surgery and reported that stereopsis, not VA, was significantly correlated with VR-QoL after first- and second-eye cataract surgeries [17]. Moreover, a population-based cross-sectional study that investigated VR-QoL and visual functions in 9330 members of the 1958 British Birth Cohort (ages 44 and 45) showed that impairment of VR-QoL was strongly associated with impaired stereopsis [18]. Based on these results and our study results, stereopsis is associated with VR-QoL and may be important in patients with CRVO.

The TST value was significantly associated with VR-QoL at baseline and not after treatment. In contrast, the TNO value was significantly correlated with VR-QoL after treatment and not at baseline. The cause of this discrepancy is unclear. However, we hypothesized that varying index sizes between the two stereotests may have contributed. The diameter of the TNO plate stimulus (8.5°) is larger than that of the TST circle stimulus (0.7°); thus, the TNO value may reflect the function of a larger area of the retina than the TST value. Before treatment, the CFT was very thick; thus, the function of the center of the macula was impaired. Therefore, the TST value was associated with VR-QoL. After treatment, the CFT decreased; thus, impairment of both the center of the macula and the entire posterior pole became apparent. Therefore, the TNO value was associated with VR-QoL after treatment.

Interestingly, changes in LCS were significantly correlated with changes in the VFQ-25 composite score, whereas changes in other visual functions were not correlated with an improvement in the VFQ-25 composite score. According to a previous study that examined the relationship between changes in VR-QoL and various visual functions before and after vitrectomy performed for various retinal diseases, the improvement in LCS was significantly correlated with improvement in the VFQ-25 composite score in patients with PDR and DME [14]. Based on these findings and our study, in some retinal disorders, including CRVO-CME, CS may be a more important visual functional parameter than VA.

Metamorphopsia and aniseikonia were not associated with the VFQ-25 composite score in the current study. Studies have reported that metamorphopsia was associated with VR-QoL in patients with ERM [7] and MH [9]. The severity of metamorphopsia in the present study was 0.2 at baseline and 0.3 at 12 months after treatment. This is lower than the severity of metamorphopsia found in the studies of patients with ERM [7] and MH [9] (0.79 at baseline, 0.44 after surgery; 0.77 at baseline, 0.45 after surgery, respectively). Aniseikonia has been found to be associated with VR-QoL in patients with ERM [8]. The mean percentage of aniseikonia was 0.1% at baseline and 0.2% at 12 months after treatment in our study. These values are lower than the 6.2% of aniseikonia in patients with ERM reported in a study by Okamoto et al. [19]. In the present study, the degrees of metamorphopsia and aniseikonia were relatively low in patients with CRVO and unlikely to affect VR-QoL.

This study had some limitations. First, our sample size was small. Second, we did not classify the patients into ischemic and non-ischemic CRVO. The effects of therapy on visual functions and VR-QoL are believed to differ between ischemic and non-ischemic CRVO. Future studies with a larger sample size and the classification of patients with ischemic or non-ischemic CRVO are warranted.

## 5. Conclusions

In conclusion, BCVA, CS, stereopsis, and VR-QoL improved after IVR in patients with CRVO-CME. The BCVA of the fellow eye was significantly correlated with VR-QoL at baseline and 12 months after treatment. Thus, the BCVA of the fellow eye had the strongest impact on VR-QoL. These results suggest that clinicians should be mindful of the condition of the fellow eye as well as that of the affected eye when treating patients with CRVO. Stereopsis was also significantly correlated with VR-QoL 12 months after treatment, and improvement in the CS of the affected eye was significantly correlated with improvement in VR-QoL. Thus, the CS of the affected eye and stereopsis are important visual functional parameters in patients with CRVO-CME. This implies that clinicians should measure CS and stereopsis in daily practice.

## Figures and Tables

**Table 1:** Baseline clinical characteristics of patients.

Number of Eyes	23
Age (years)	72.2 ± 11.1
Sex (men/women)	13/10
BCVA of the affected eye at baseline (logMAR)	0.79 ± 0.56
LCS of the affected eye at baseline (letters)	7.7 ± 7.7
Severity of metamorphopsia of the affected eye at baseline (degree)	0.2 ± 0.3
Amount of aniseikonia at baseline (%)	0.1 ± 2.6
Titmus Stereo Test value at baseline (log)	3.2 ± 0.7
TNO stereotest value at baseline (log)	3.3 ± 0.6
BCVA of the fellow eye (logMAR)	0.01 ± 0.13
LCS of the fellow eye (letters)	21.1 ± 3.3
Central foveal thickness at baseline (µm)	771 ± 319

Data are presented as number or mean ± standard deviation; BCVA, best-corrected visual acuity; logMAR, logarithm of the minimum angle of resolution; LCS, letter contrast sensitivity.

**Table 2:** Visual functions and CFT at baseline and 3, 6, and 12 months after treatment.

	Baseline	3 M	6 M	12 M
Best-corrected visual acuity (logMAR)	0.79 ± 0.56	0.43 ± 0.49	0.51 ± 0.51	0.59 ± 0.61
*p* value (from preoperative logMAR BCVA)		0.001 ***	0.005 **	0.038 *
LCS (letters)	7.7 ± 7.7	14.4 ± 7.6	13.0 ± 7.4	12.4 ± 9.0
*p* value (from LCS at baseline)		0.001 ***	0.001 ***	0.031 *
Severity of metamorphopsia (degree)	0.2 ± 0.3	0.3 ± 0.6	0.4 ± 0.5	0.3 ± 0.5
*p* value (from severity of metamorphopsia at baseline)		0.203	0.247	0.582
Amount of aniseikonia (%)	0.1 ± 2.6	−0.6 ± 1.6	−1.0 ± 2.3	0.2 ± 0.9
*p* value (from amount of aniseikonia at baseline)		0.292	0.283	0.138
TST value (log)	3.2 ± 0.7	2.7 ± 0.8	2.7 ± 0.8	2.7 ± 0.8
*p* value (from TST value at baseline)		0.001 ***	0.008 **	0.023 *
TNO stereotest value (log)	3.3 ± 0.6	2.9 ± 0.7	2.9 ± 0.7	2.8 ± 0.8
*p* value (from TNO stereotest value at baseline)		0.003 ***	0.009 **	0.012 *
VFQ-25 composite score	62.6 ± 16.9	70.6 ± 15.6	72.2 ± 15.8	74.3 ± 14.6
*p* value (from VFQ-25 composite score at baseline)		0.003 ***	0.002 ***	0.002 **
CFT (µm)	771 ± 319	181 ± 58	361 ± 303	308 ± 259
*p* value (from CFT at baseline)		0.001 ***	0.001 ***	0.001 ***

Data are presented as mean ± standard deviation; M, months after treatment; LCS, letter contrast sensitivity; TST, Titmus Stereo Test; VFQ-25, the 25-item National Eye Institute Visual Function Questionnaire; CFT, central foveal thickness; * significant correlations between parameters (Wilcoxon’s signed-rank test, *** *p* < 0.005, ** *p* < 0.01, * *p* < 0.05).

**Table 3:** Visual functions affecting VFQ-25 composite score at baseline.

	Simple Regression Analysis		Multiple Regression Analyses	
	R	*p*	Standard β	*p*
BCVA of the affected eye	−0.184	0.412	0.126	0.576
BCVA of the fellow eye	−0.459	0.032 *	−0.568	0.011 ^†^
LCS of the affected eye	0.303	0.181	0.038	0.871
LCS of the fellow eye	0.364	0.115	0.138	0.671
Severity of metamorphopsia of the affected eye	−0.047	0.835	−0.076	0.182
Amount of aniseikonia	0.268	0.229	−0.271	0.182
Titmus Stereo Test value	−0.450	0.036 *	−0.149	0.515
TNO stereotest value	−0.419	0.052	−0.057	0.815

VFQ-25, the 25-item National Eye Institute Visual Function Questionnaire; BCVA, best-corrected visual acuity; LCS, letter contrast sensitivity; * significant correlations between parameters (Spearman’s rank coefficient, * *p* < 0.05); ^†^ significant correlations between parameters (stepwise multiple regression analyses, ^†^ *p* < 0.05).

**Table 4:** Visual functions affecting VFQ-25 composite score 12 months after treatment.

	Simple Regression Analysis		Multiple Regression Analyses	
	R	*p*	Standard β	*p*
BCVA of the affected eye	−0.554	0.021 *	0.076	0.772
BCVA of the fellow eye	−0.687	0.002 **	−0.527	0.005 ^††^
LCS of the affected eye	0.517	0.033 *	0.230	0.580
LCS of the fellow eye	0.579	0.015 *	−0.016	0.950
Severity of metamorphopsia of the affected eye	−0.223	0.390	−0.224	0.142
Amount of aniseikonia	0.288	0.263	−0.294	0.075
Titmus Stereo Test value	−0.419	0.094	0.209	0.468
TNO stereotest value	−0.660	0.004 **	−0.519	0.01 ^†^

VFQ-25, the 25-item National Eye Institute Visual Function Questionnaire; BCVA, best-corrected visual acuity; LCS, letter contrast sensitivity; * significant correlations between parameters (Spearman’s rank coefficient, ** *p* < 0.005, * *p* < 0.05); ^†^ significant correlations between parameters (stepwise multiple regression analyses, ^††^ *p* < 0.01, ^†^ *p* < 0.05).

**Table 5:** Simple regression analysis between changes in the VFQ-25 composite score and changes in several visual functions.

	Changes in the VFQ-25 Composite Score	
	R	*p*
Changes in best-corrected visual acuity	−0.068	0.796
Changes in letter contrast sensitivity	0.505	0.039 *
Changes in severity of metamorphopsia	0.126	0.629
Changes in amount of aniseikonia	0.021	0.937
Changes in Titmus Stereo Test value	−0.443	0.075
Changes in TNO stereotest value	−0.443	0.075

VFQ-25, the 25-item National Eye Institute Visual Function Questionnaire; * significant correlations between parameters (Spearman’s rank coefficient, * *p* < 0.05).

## Data Availability

Study data are available from the corresponding author on reasonable request.
